# Public health emergency due to rains and floods in Rio Grande do Sul, Brazil: an ecological study, 2024

**DOI:** 10.1590/1980-549720260003

**Published:** 2026-01-30

**Authors:** Ana Sara Semeão de Souza, João Roberto Cavalcante, Paula Orofino Moura Costa, Maiara Almeida Maia, Raquel Proença, Carlos Henrique Michiles Frank, Alessandro Pecego Martins Romano, Thiago Basílio Mendonça, Cristilene Delfino, José Lucas Pinho da Fonseca, Daniel Roberto Coradi de Freitas, Edenilo Baltazar Barreira, Ethel Leonor Maciel, Márcio Henrique de Oliveira Garcia

**Affiliations:** I Ministry of Health, Secretariat of Health Surveillance and Environment, Department of Public Health Emergencies – Brasília (DF), Brazil.; II Universidade do Estado do Rio de Janeiro, Institute of Social Medicine – Rio de Janeiro (RJ), Brazil.; III Ministry of Health, Secretariat of Health Surveillance and Environment – Brasília (DF), Brazil.

**Keywords:** Surge capacity, Natural disasters, Surveillance in disasters, Declaration of emergency, Public health

## Abstract

**Objective:**

To characterize the public health impacts resulting from the natural disaster caused by heavy rainfall and flooding in the state of Rio Grande do Sul (RS), Brazil, in 2024.

**Methods:**

This was a descriptive ecological study covering the period between April 29 and May 31, 2024. The units of analysis were the municipalities and the nine functional planning regions. The following were analyzed: number and proportion of municipalities with an emergency declaration; deaths, ill individuals, and injured individuals per 100,000 inhabitants; and displaced and homeless people per thousand inhabitants.

**Results:**

Of the 497 municipalities in RS, 91.3% declared an emergency, with emphasis on regions R2 and R5, where all municipalities were affected. Regions R1 and R2 were the most impacted in terms of human harm, particularly regarding homelessness, reaching 116 and 92.9 per thousand inhabitants, respectively. Emergencies were more frequent in regions R6, R7, and R5. The spatial distribution reveals that the most affected municipalities are geographically close, especially in regions R1, R2, and R8.

**Conclusion:**

The unequal impacts across regions highlight the urgency of territorialized public policies, focused on prevention and emergency response. Strengthening risk governance and public health must occupy a central position in the climate agenda, promoting equity and resilience in the face of disasters.

## INTRODUCTION

Heavy rainfall and flooding are among the most prevalent natural disasters worldwide and constitute a major source of material and human losses, disproportionately impacting populations with lower socioeconomic status^1,2^. Between 1990 and 2022, more than 4,000 flood events were documented across 168 countries and territories, affecting over 3.2 billion individuals and causing more than 218,000 deaths. These events generated an estimated US$ 1.3 trillion in economic losses^3^, in addition to producing enduring effects on public health systems and the social determinants of health.

In 2023, the Southern Region of Brazil reported three major emergencies related to intense rainfall and flooding between June and November, two of which were associated with extratropical cyclones^4^. In the last week of April 2024, the state of Rio Grande do Sul (RS), located in the Southern Region of Brazil, experienced unprecedented heavy rainfall. The region had been under monitoring since April 26, following alerts issued by the National Center for Risk and Disaster Management of the Ministry of Regional Integration and Development^5^.

Floods devastated a large portion of the state's territory. Numerous municipalities were affected, along with roadways, communication networks, health services, and electricity and water supply systems^6^. Families impacted by the floods face challenges such as displacement, food insecurity, unexpected health problems, worsening of chronic conditions, heightened risk of communicable disease outbreaks, and financial hardship, all of which will necessitate an extended recovery period^7^.

In this context, strengthening surveillance, prevention, and mitigation strategies tailored to regional conditions becomes increasingly necessary, with emphasis on reinforcing the infrastructure of the Brazilian Unified Health System (*Sistema Único de Saúde* – SUS) and expanding the public health response capacity. The aim is to support more equitable and context-specific interventions during such emergencies. This study sought to characterize the natural disaster caused by intense rainfall and flooding in the state of Rio Grande do Sul in 2024.

## METHODS

This descriptive ecological study examined the natural disaster caused by heavy rainfall and flooding in the state of RS, covering the period from April 29 to May 31, 2024. The analysis period spans the onset of the heavy rains through the end of the flooding, which lasted approximately one month^5^. The units of analysis were the municipalities and the nine functional planning regions. These functional planning regions were defined based on criteria of economic, environmental, and social homogeneity, as well as variables related to employment hubs, commuting patterns by type of transport, urban hierarchy, and the organization of health and higher education service networks, among other factors^8^.

**Figure 1:**
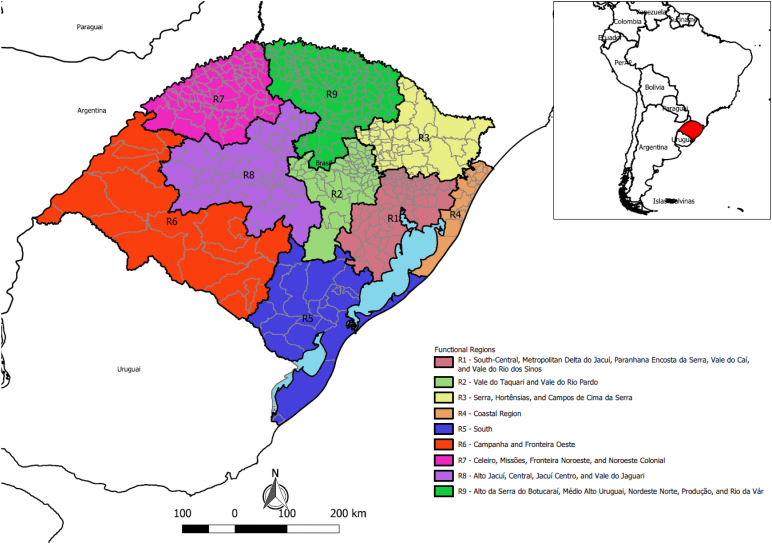
Location of the territory and division of the state of Rio Grande do Sul by functional planning regions. Rio Grande do Sul, 2024.

According to the Brazilian Institute of Geography and Statistics (*Instituto Brasileiro de Geografia e Estatística* – IBGE), the state of RS comprises 497 municipalities (8.9% of all municipalities in Brazil), organized into nine functional planning regions^8,9^. It is the third-largest state in the country in terms of territorial area (281,707.15 km^2^). In 2022, its population was estimated at 10,882,965 inhabitants — representing the sixth-largest population nationally, with a population density of 38.6 inhabitants per km^2^ (Figure 1).

The following data sources were used: reports published on the Civil Defense/RS page^10^; the management report on reported damages from the Integrated Disaster Information System^11^; and the digital mesh of functional regions available on the State Spatial Data Infrastructure portal of Rio Grande do Sul^12^.

The variables analyzed included: the number and proportion of municipalities with a declaration of emergency (state of public calamity or emergency situation) recognized by Decree 57.646^13^ of May 31, 2024, or by individual municipal decrees^10^; deaths, illnesses, and injuries per 100,000 inhabitants; and displaced and homeless individuals per 1,000 inhabitants^11^, according to functional planning regions. The total population of each functional planning region was obtained by summing the populations of its constituent municipalities. Municipal population data provided by the IBGE, based on the 2023 intercensal estimates, were used.

A State of Emergency is declared when adverse events, whether natural or human-made, result in damage and losses that partially compromise the response capacity of the public authorities of the affected federative entity. A State of Public Calamity is declared when the magnitude of the disaster is sufficiently severe that the resulting damage and losses exceed the response capacity of the public authorities of the affected federative entity^5^.

Thematic maps were created to assess the spatial distribution of the analyzed variables. The maps were generated using QGIS 2.18.6 software, employing the administrative-political map of the state of RS with municipal boundaries (in shapefile format — shp.) obtained from IBGE^10^, along with the functional planning regions^12^.

In accordance with local ethical regulations, this study did not require ethical approval. The data were obtained from publicly available secondary sources and did not include sensitive or confidential information that might infringe upon personal data protection rights.

### Data availability statement:

The entire dataset supporting the finding of this study is available upon request to the corresponding author.

## RESULTS

Of the 497 municipalities in RS, 91.3% declared a state of emergency due to heavy rainfall and flooding. Of these, 395 (79.5%) declared a state of emergency and 95 (19.1%) declared a state of public calamity. Across the functional planning regions, all municipalities in regions R2 and R5 declared some type of emergency. Regions R4 and R9 recorded the lowest proportions of municipalities declaring an emergency, at 71.4 and 82.3%, respectively (Table 1).

**Table 1:** Municipalities with emergency declarations according to functional planning regions. Rio Grande do Sul, 2024.

Functional Planning Regions	Municipalities	Emergency Situation	Public Calamity Status
	N (%)	N (%)	N (%)
R1	70 (14.1)	42 (60.0)	27 (38.6)
R2	59 (11.9)	24 (40.7)	35 (59.3)
R3	49 (9.9)	37 (75.5)	8 (16.3)
R4	21 (4.2)	14 (66.7)	1 (4.8)
R5	22 (4.4)	18 (81.8)	4 (18.2)
R6	20 (4.0)	19 (95.0)	0
R7	77 (15.5)	71 (92.2)	0
R8	49 (9.9)	32 (65.3)	15 (30.6)
R9	130 (26.2)	102 (78.5)	5 (3.8)
Total	497 (100)	395 (72.2)	95 (19.1)

Regarding the state of public calamity, region R2 showed the highest proportion of municipalities in this condition (59.3%), followed by R1 (38.6%) and R8 (30.6%). In contrast, regions R6 (95%), R7 (92.2%), and R5 (81.8%) presented the highest proportions of municipalities declaring a state of emergency without reaching the level of public calamity. Although the severity of damage was greatest in R2, R1, and R8, other regions, such as R6 and R7, were also substantially affected, albeit to a lesser degree (Table 1).

Region R2 presented the highest mortality rate (5.2 deaths/100,000 inhabitants), more than twice the rate observed in region R3 (2.7/100,000). With respect to the sick or injured population, regions R1 and R2 showed the highest rates. In R1, 68.0 sick individuals and 184.0 injured individuals per 100,000 inhabitants were recorded, values that exceeded those reported in R2 (33.9 and 66.0/100,000, respectively) (Table 2).

**Table 2:** Human damages resulting from the emergency caused by heavy rainfall and flooding, by functional planning regions. Rio Grande do Sul, 2024.

Functional Planning Regions	Deaths per 100,000 inhab.	Ill persons per 100,000 inhab.	Injured per 100,000 inhab.	Displaced per 1,000 inhab.	Homeless per 1,000 inhab.
R1	0.3	68.0	184.0	14.8	92.9
R2	5.2	33.9	66.0	14.6	116.3
R3	2.7	0.8	12.5	1.4	7.0
R4	0	0	2.3	0.8	50.3
R5	0	0	0	1.4	4.6
R6	0	0	0	1.2	8.7
R7	0	0	0	0.2	1.9
R8	1.1	3.1	6.1	2.6	11.1
R9	0.1	6.6	12.0	1.0	4.9
Total	0.9	30.8	81.8	7.7	50.0

Regarding displaced persons, regions R1 and R2 recorded the highest proportions, with approximately 15 individuals per 1,000 inhabitants, characterizing them as areas of greater housing instability. With respect to homelessness, region R2 again presented the most critical situation, with approximately 116 individuals per 1,000 inhabitants, followed by R1 (92.9/1,000) and R4 (50.3/1,000) (Table 2).

**Figure 2:**
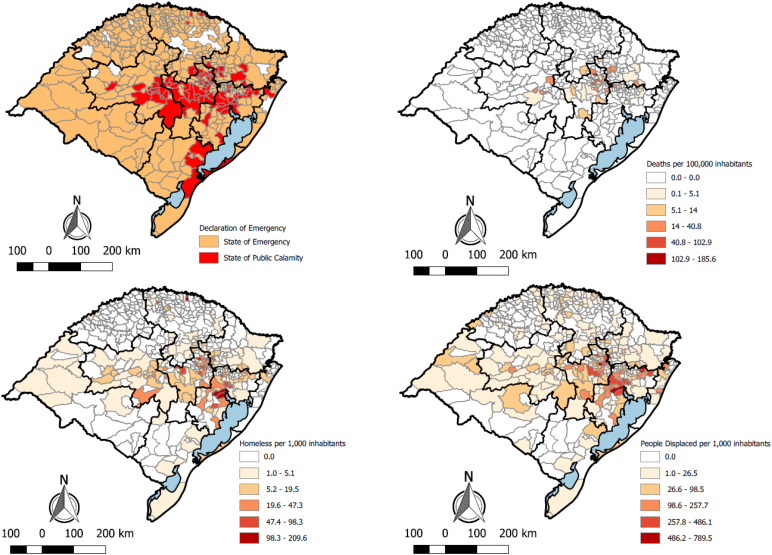
(A) Municipalities with a declaration of emergency, (B) deaths per 100,000 inhabitants, (C) homeless and (D) displaced per 1,000 inhabitants. Rio Grande do Sul, 2024

The declaration of a state of public calamity was predominantly concentrated in regions R8, R1, and R2, whose municipalities are geographically contiguous (Figure 2A). Region R2 showed the greatest impact in terms of human losses due to deaths, with a relatively homogeneous distribution across its municipalities (Figure 2B). Regions R1 and R2 also exhibited the highest per capita rates of homeless individuals, with affected municipalities located in close proximity to one another (Figure 2C). Although the municipalities with the highest numbers of homeless individuals per capita were found in regions R1 and R2, their spatial distribution within these regions was more dispersed (Figure 2D).

## DISCUSSION

The high proportion of municipalities declaring a state of emergency or public calamity (91.3%) indicates widespread vulnerability in the state of RS, particularly in functional planning regions R2 and R1, which concentrated the most severe human and structural impacts. These two regions presented the poorest indicators evaluated: higher mortality, greater numbers of sick and injured individuals, and the highest rates of displaced and homeless persons.

The vulnerability scenario initially identified in the state of RS manifests differently in functional planning regions R1 and R2. Region R1, characterized by intense urbanization, socioeconomic polarization, and unplanned growth, concentrates problems typical of large metropolitan areas, which exacerbate the human and structural impacts of emergencies. In contrast, region R2 exhibits vulnerabilities associated with a predominantly rural population, demographic aging, and deficiencies in basic sanitation^14,15^. Thus, the results of the evaluated indicators reflect not only the severity of the event but also the distinct structural vulnerabilities of each region, underscoring the need for differentiated preparedness and response strategies.

The widespread use of effective early warning systems for hydrological events has contributed to the reduction in flood-related mortality^2^. However, areas in which populations are substantially affected require comprehensive and well-designed flood risk-reduction strategies to mitigate the associated impacts. Consequently, increased investment and resource redistribution are necessary to strengthen flood prevention measures and enhance emergency response capacities^1,3^.

Furthermore, the magnitude and extent of the public health emergency indicate a substantial burden on local health and social care services. Several facilities, including hospitals, Emergency Care Units (*Unidades de Pronto Atendimento* – UPA), and Basic Health Units (*Unidades Básicas de Saúde* – UBS), experienced partial or total service disruptions, resulting not only from physical damage to infrastructure but also from the absence of health professionals directly affected by the floods^16,17^. The interruption of essential services compromises both local and regional response capacity and underscores the need to incorporate resilience strategies into public health policies. Strengthening the adaptive capacity of SUS, particularly in territories with heightened vulnerability, is essential for mitigating harm and preserving the comprehensiveness of care in crisis situations.

The geographical continuity among the most affected municipalities (particularly in regions R1, R2, and R8) indicates that the impacts did not occur in isolation but instead affected entire regions in an interdependent manner, thereby requiring coordinated responses among neighboring municipalities. The development and implementation of Climate Change Adaptation Plans, as well as the construction of Contingency Plans, constitute essential strategies for reducing the risks and impacts associated with extreme weather events and climate change, as well as the disasters they trigger^5,18,19^.

These plans can strengthen the adaptive capacity of cities, contributing to the prevention of deaths, the mitigation of infrastructure damage, and the reduction of material losses. By promoting integrated preparedness and response measures, such instruments are essential for protecting population health and enhancing the resilience of territories in the face of disasters.

The effects of flood events are often subject to approximations and aggregations that carry a high degree of inaccuracy. Although the availability and quality of data have improved over time, no records exist of experiences with an integrated disaster information system, which has resulted in the increased use of multiple data sources for reporting. Consequently, in many events, the frequency of injuries and the number of affected individuals remain unknown, unreported, or difficult to identify. For other outcomes, such as deaths, reporting frequently lacks essential details, which likely contributes to the substantial underestimation of the impacts of flood events on human populations^3^.

The use of indicators such as deaths, injuries, illnesses, homelessness, and displacement per capita enables the assessment of disaster impacts and offers a precise basis for mitigation, preparedness, and response actions, in addition to supporting regional health planning. However, understanding the sociodemographic characteristics of the affected population can enhance the organization and planning of recovery efforts that promote equity.

A recent study assessed whether response actions during a public health emergency incorporate information on social determinants into decision-making with the aim of reducing inequalities^20^. The results identified three gaps in the decision-making and guidance processes during the response: insufficient data to support equitable decision-making, persistence of pre-existing health inequalities, and the absence of tools designed to guide decisions toward reducing inequalities^20^.

In this context, the gaps identified regarding the incorporation of social determinants into public health emergency response actions are closely aligned with the results observed in RS. The high proportion of municipalities declaring a state of emergency or public calamity, together with the indicators of mortality, illness, and population displacement, reflects inequalities among functional regions that cannot be fully explained by quantitative impact data alone. The limited integration of social information into monitoring and decision-making systems constrains the formulation of equitable and sustainable responses, thereby contributing to the persistence of pre-existing inequalities. Thus, the systematic incorporation of social determinants of health into emergency planning and management is essential for territorialized public policies to not only reduce the immediate effects of disasters but also address, in a structured manner, the disparities that intensify their impacts.

Recent studies indicate that public health emergencies related to flooding are likely to increase in the coming years^3,7^, underscoring the need for greater emphasis on strategies for recording information about these events. Such strategies aim not only to improve future emergency responses based on lessons learned but also to enable faster and more effective information management during events, with a focus on equity. The fragmentation of information across systems, as well as the limited capacity to capture strategic data during disasters, highlights a critical challenge that must be addressed in Brazil in the coming years.

The fact that countries with greater resources are better able to predict and respond to imminent flood events^2^ demonstrates that the development of information systems and the capacity for timely detection and response to such disasters should be prioritized in less developed countries. Changes in socioeconomic, demographic, geological, and climatological conditions indicate not only that floods will become more frequent, but also that their impacts on human populations will be increasingly significant in the coming decades^1,3,7^.

Finally, the differences observed among the functional planning regions regarding the magnitude of impacts underscore the need for territorially based public policies that emphasize prevention, rapid response, and the strengthening of health infrastructure and social protection in the most vulnerable areas. This context highlights the urgent need for stronger governance in disaster risk management, including more effective flood mitigation strategies, improved emergency systems, and international support, particularly for the reconstruction of affected areas after disasters. In addition, integrated strategies that consider the effects of climate change and environmental degradation on public health are essential, reinforcing the importance of a proactive and sustainable approach to disaster risk reduction.

Given the observed impacts, it is evident that public health emergencies caused by floods require a paradigm shift in risk management and public policy formulation in Brazil. In this context, the events analyzed must be understood within the broader global ecological crisis, which has intensified the frequency and severity of natural disasters and imposed increasing challenges on public health and the resilience of SUS. Beyond strengthening existing structures, it is necessary to advance toward building a culture of prevention, emphasizing preparedness, adequate resource allocation, and coordinated action across different levels of government. Moreover, integrating disaster surveillance into the public health agenda, while incorporating dimensions of environmental justice and equity, can enhance the effectiveness of prevention and response measures and reduce territorial inequalities in access to protection and care.

It is worth noting that the ecological approach adopted in this study, while enabling the identification of relevant spatial patterns and supporting the planning of public health actions, presents limitations, particularly in assessing individual determinants of vulnerability and socioeconomic inequalities within the analyzed regions. An effective disaster response must be anchored in qualified information systems capable of guiding real-time decisions and reducing inequalities in access to protection and care. The vulnerability exposed by this emergency reinforces that public health must assume a central role in the climate agenda, coordinating short-, medium-, and long-term actions to ensure the continuity of health services and promote social justice in crisis contexts.
