# Supplementary material for: Public health emergency due to rains and floods in Rio Grande do Sul, Brazil: an ecological study, 2024
Source: Rev Bras Epidemiol. 2026 Jan 30;29:e260003. doi: 10.1590/1980-549720260003 (PMC12858087; doi:10.1590/1980-549720260003)
Supplement: Translation

# Translation: Emergência em saúde pública por chuvas e inundações no Rio Grande do Sul, Brasil: um estudo ecológico, 2024

**DOI:** 10.1590/1980-549720260003.2

**Authors:** Ana Sara Semeão de Souza, João Roberto Cavalcante, Paula Orofino Moura Costa, Maiara Almeida Maia, Raquel Proença, Carlos Henrique Michiles Frank, Alessandro Pecego Martins Romano, Thiago Basílio Mendonça, Cristilene Delfino, José Lucas Pinho da Fonseca, Daniel Roberto Coradi de Freitas, Edenilo Baltazar Barreira, Ethel Leonor Maciel, Márcio Henrique de Oliveira Garcia

**Affiliations:** I Ministério da Saúde, Secretaria de Vigilância em Saúde e Ambiente, Departamento de Emergências em Saúde Pública – Brasília (DF), Brazil.; II Universidade do Estado do Rio de Janeiro, Instituto de Medicina Social – Rio de Janeiro (RJ), Brazil.; III Ministério da Saúde, Secretaria de Vigilância em Saúde e Ambiente – Brasília (DF), Brazil.

**Keywords:** Capacidade de resposta ante emergências, Desastres naturais, Vigilância em desastres, Declaração de estado de emergência em desastres, Saúde pública

## Abstract

**Objetivo:**

Caracterizar os impactos em saúde pública decorrentes do desastre natural ocasionado por chuvas intensas e inundações no estado do Rio Grande do Sul (RS), no Brasil, em 2024.

**Métodos:**

Trata-se de um estudo ecológico descritivo, compreendendo o período entre 29 de abril e 31 de maio de 2024. As unidades de análise foram os municípios e as nove regiões funcionais de planejamento. Foram analisados: número e proporção de municípios com declaração de emergência; óbitos, enfermos e feridos por 100 mil habitantes; e desalojados e desabrigados a cada mil habitantes.

**Resultados:**

Dos 497 municípios do RS, 91,3% declararam emergência, com destaque para as regiões R2 e R5, onde todos os municípios foram afetados. As regiões R1 e R2 foram as mais impactadas em termos de danos humanos, com destaque para os desabrigados, chegando a 116 e 92,9 por mil habitantes, respectivamente. A situação de emergência foi mais frequente nas regiões R6, R7 e R5. A distribuição espacial revela que os municípios mais afetados estão geograficamente próximos, especialmente nas regiões R1, R2 e R8.

**Conclusão:**

Os impactos desiguais entre as regiões ressaltam a urgência de políticas públicas territorializadas, com foco na prevenção e resposta às emergências. Fortalecer a governança de riscos e a saúde pública deve ocupar posição central na agenda climática, promovendo equidade e resiliência diante dos desastres.

## INTRODUÇÃO

As chuvas intensas e inundações são os desastres naturais mais comuns em todo o mundo, sendo uma das principais causas de danos materiais e humanos que afetam desproporcionalmente populações com baixa condição socioeconômica^1,2^. De 1990 a 2022, foram registrados mais de 4 mil eventos de enchentes em 168 países e territórios, que afetaram mais de 3,2 bilhões de pessoas e resultaram em mais de 218 mil mortes. Esses eventos causaram US$ 1,3 trilhão em prejuízos econômicos^3^, além de gerar impactos duradouros sobre os sistemas de saúde pública e sobre os determinantes sociais da saúde.

Em 2023, a Região Sul do Brasil notificou três emergências de grande porte por chuvas intensas e inundações entre os meses de junho e novembro, sendo dois desses eventos causados por ciclones extratropicais^4^. Na última semana de abril de 2024, o Rio Grande do Sul (RS), estado localizado na Região Sul do Brasil, enfrentou chuvas intensas sem precedentes. A região estava sendo monitorada desde o dia 26 de abril por conta dos alertas emitidos pelo Centro Nacional de Gerenciamento de Riscos e Desastres do Ministério da Integração de Desenvolvimento Regional^5^.

As inundações devastaram grande parte da extensão territorial do estado. Vários municípios foram atingidos pelas inundações, bem como estradas, redes de comunicação, serviços de saúde e sistemas de abastecimento de energia elétrica e de água^6^. As famílias afetadas pelas inundações enfrentam desafios como o deslocamento, insegurança alimentar, problemas de saúde inesperados, agravamento de condições crônicas e maior risco de surtos de doenças transmissíveis e dificuldades financeiras, o que demandará um tempo prolongado de recuperação^7^.

Nesse sentido, torna-se cada vez mais necessário fortalecer estratégias de vigilância, prevenção e mitigação adaptadas às realidades regionais, com ênfase no fortalecimento da infraestrutura do Sistema Único de Saúde (SUS) e na ampliação da capacidade de resposta em saúde pública, visando respostas a estas emergências com intervenções mais equitativas e contextualizadas. O presente estudo buscou caracterizar o desastre natural ocasionado por chuvas intensas e inundações no estado do Rio Grande do Sul, em 2024.

## MÉTODOS

Trata-se de um estudo ecológico descritivo do desastre natural devido às chuvas intensas e inundações no estado do RS, compreendendo o período entre 29 de abril e 31 de maio de 2024. O período de análise abrange o início das chuvas intensas até o fim das inundações, que duraram cerca de um mês^5^. As unidades de análise foram os municípios e as nove regiões de funcionais de planejamento. As regiões funcionais de planejamento foram propostas considerando-se critérios de homogeneidade econômica, ambiental e social e em variáveis relacionadas à identificação das polarizações de emprego, dos deslocamentos por tipo de transporte, da hierarquia urbana, da organização da rede de serviços de saúde e educação superior, entre outros^8^.

Segundo o Instituto Brasileiro de Geografia e Estatística (IBGE), o estado do RS possui 497 municípios (8,9% do total de municípios do Brasil), divididos em nove regiões funcionais de planejamento^8,9^. É o terceiro maior estado do país em dimensão territorial (281.707,15 km^2^). Em 2022, sua população foi estimada em 10.882.965 de habitantes — a sexta maior população do país, com densidade demográfica de 38,6 habitantes por km^2^ (Figura 1).

**Figura 1:** Localização do território e divisão do estado do Rio Grande do Sul por regiões funcionais de planejamento. Rio Grande do Sul, 2024.

Foram utilizadas como fontes de dados: os relatórios publicados na página da Defesa Civil/RS^10^; o relatório gerencial de danos informados do Sistema Integrado de Informações sobre Desastres^11^; e a malha digital das regiões funcionais disponibilizada no portal de Infraestrutura Estadual de Dados Espaciais — RS^12^.

As variáveis analisadas foram: número e proporção de municípios com declaração de emergência (estado de calamidade pública e situação de emergência) reconhecida pelo Decreto 57.646^13^, de 31 de maio de 2025, ou por decretos individuais^10^; óbitos, enfermos e feridos por 100 mil habitantes; e desalojados e desabrigados a cada mil habitantes^11^, segundo regiões funcionais de planejamento. A população total de cada região funcional de planejamento foi obtida por meio da soma das populações dos municípios que a compõem. Foram utilizados os dados populacionais de cada município, disponibilizados pelo IBGE, referentes às estimativas intercensitárias de 2023.

A Situação de Emergência é declarada quando eventos adversos, sejam naturais, sejam provocados pelo homem, causam danos e prejuízos que comprometem, de forma parcial, a capacidade de resposta do poder público do ente federativo afetado. O Estado de Calamidade Pública é declarado quando a magnitude do desastre é tão grande que os danos e prejuízos causados ultrapassam a capacidade de resposta do poder público do ente federativo afetado^5^.

Mapas temáticos foram construídos para a avaliação da distribuição espacial das variáveis analisadas. Os mapas foram construídos por meio do *software* QGis 2.18.6, utilizando o mapa administrativo-político com divisão dos municípios do estado do RS (em formato *shapefile* — shp.) extraídos do IBGE^10^ e das regiões funcionais de planejamento^12^.

Conforme as regulamentações éticas locais, este estudo não exigiu aprovação ética. Os dados foram obtidos de registros secundários que estão disponíveis para acesso público e não contêm nenhuma informação sensível ou confidencial que viole os direitos à proteção de dados pessoais.

### Declaração de disponibilidade de dados:

Todo o conjunto de dados que dá suporte aos resultados deste estudo foi publicado no próprio artigo.

## RESULTADOS

Dos 497 municípios do RS, 91,3% declararam emergência em decorrência das chuvas intensas e inundações; destes, 395 (79,5%) estavam em situação de emergência e 95 (19,1%) em estado de calamidade pública. Com relação às regiões funcionais, todos os municípios da R2 e da R5 declararam algum tipo de emergência. As regiões R4 e R9 foram as que apresentaram menor proporção de municípios com declaração de emergência, sendo 71,4 e 82,3%, respectivamente (Tabela 1).

**Tabela 1:** Municípios com declaração de emergência segundo regiões funcionais de planejamento. Rio Grande do Sul, 2024.

Regiões Funcionais de Planejamento	Municípios	Situação de Emergência	Estado de Calamidade Pública
	N (%)	N (%)	N (%)
R1	70 (14,1)	42 (60,0)	27 (38,6)
R2	59 (11,9)	24 (40,7)	35 (59,3)
R3	49 (9,9)	37 (75,5)	8 (16,3)
R4	21 (4,2)	14 (66,7)	1 (4,8)
R5	22 (4,4)	18 (81,8)	4 (18,2)
R6	20 (4,0)	19 (95,0)	0
R7	77 (15,5)	71 (92,2)	0
R8	49 (9,9)	32 (65,3)	15 (30,6)
R9	130 (26,2)	102 (78,5)	5 (3,8)
Total	497 (100)	395 (72,2)	95 (19,1)

No que se refere ao estado de calamidade pública, destaca-se a R2, com 59,3% dos municípios nessa condição, seguida da R1 (38,6%) e da R8 (30,6%). Por outro lado, as regiões R6 (95%), R7 (92,2%) e R5 (81,8%) concentraram as maiores proporções de municípios em situação de emergência, sem atingir o patamar de calamidade. Embora a intensidade dos danos tenha sido mais crítica na R2, R1 e R8, outras regiões, como R6 e R7, também foram amplamente impactadas, ainda que em menor gravidade (Tabela 1).

A região R2 apresentou a maior mortalidade (5,2 óbitos/100 mil habitantes), mais que o dobro da taxa observada na R3 (2,7/100 mil). Com relação à população enferma ou ferida, tanto a R1 quanto a R2 concentraram as maiores taxas: na R1 foram notificadas 68,0 pessoas enfermas e 184,0 feridas por 100 mil habitantes, valores superiores aos da R2 (33,9 e 66,0/100 mil, respectivamente) (Tabela 2).

**Tabela 2:** Danos humanos resultante da emergência por chuvas intensas e inundações segundo regiões funcionais de planejamento. Rio Grande do Sul, 2024.

Regiões Funcionais de Planejamento	Óbitos p/ 100.000 hab.	Enfermos p/ 100.000 hab.	Feridos p/ 100.000 hab.	Desalojados p/ 1.000 hab.	Desabrigados p/ 1.000 hab.
R1	0,3	68,0	184,0	14,8	92,9
R2	5,2	33,9	66,0	14,6	116,3
R3	2,7	0,8	12,5	1,4	7,0
R4	0	0	2,3	0,8	50,3
R5	0	0	0	1,4	4,6
R6	0	0	0	1,2	8,7
R7	0	0	0	0,2	1,9
R8	1,1	3,1	6,1	2,6	11,1
R9	0,1	6,6	12,0	1,0	4,9
Total	0,9	30,8	81,8	7,7	50,0

Quanto aos desalojados, as regiões R1 e R2 registraram as maiores proporções de desalojados, com cerca de 15 pessoas por mil habitantes, configurando-se como áreas de maior instabilidade habitacional. Já com relação aos desabrigados, novamente R2 assumiu a condição mais crítica, com aproximadamente 116 pessoas por mil habitantes, seguida da R1 (92,9/1000) e da R4 (50,3/1000) (Tabela 2).

A declaração de calamidade pública concentrou-se principalmente nas regiões R8, R1 e R2, cujos municípios apresentam continuidade geográfica (Figura 2A). A região R2 apresenta o maior impacto de danos humanos relativos a óbitos, com distribuição homogênea entre os municípios (Figura 2B). Já a R1 e a R2 apresentaram as maiores taxas de desabrigados por habitantes, com municípios espacialmente próximos (Figura 2C). Os municípios com maior número de desabrigados por habitante estão nas regiões R1 e R2, mas com distribuição espacial mais dispersa dentro da região (Figura 2D).

**Figura 2:** (A) Municípios com declaração de emergência, (B) óbitos por 100 mil habitantes, (C) desabrigados e (D) desalojados a cada mil habitantes. Rio Grande do Sul, 2024

## DISCUSSÃO

A elevada proporção de municípios em situação de emergência ou estado de calamidade pública (91,3%) revela uma vulnerabilidade generalizada no estado do RS, com destaque para as regiões funcionais de planejamento R2 e R1, que concentraram os maiores impactos humanos e estruturais. Essas duas regiões apresentaram os piores indicadores avaliados: maior mortalidade, maior número de enfermos e feridos, além das maiores taxas de desalojados e desabrigados.

O cenário de vulnerabilidade identificado inicialmente no estado do RS materializa-se de forma distinta nas regiões funcionais de planejamento R1 e R2: enquanto a R1, marcada pela intensa urbanização, pela polarização socioeconômica e pelo crescimento desordenado, concentra problemas típicos de grandes metrópoles que potencializam os impactos humanos e estruturais das emergências, a R2 apresenta fragilidades relacionadas ao predomínio da população rural, ao envelhecimento demográfico e às deficiências em saneamento básico^14,15^. Dessa forma, os resultados dos indicadores avaliados refletem não apenas a intensidade do evento, mas também as vulnerabilidades estruturais específicas de cada região, reforçando a necessidade de estratégias diferenciadas de preparação e resposta.

O uso generalizado de métodos eficazes de alerta precoce para eventos hidrológicos tem contribuído para o declínio da mortalidade por inundações^2^. No entanto, áreas onde as populações são substancialmente afetadas exigem estratégias abrangentes e bem planejadas de redução de riscos de inundações para mitigar seus efeitos. Desse modo, são necessários maior investimento e a redistribuição de recursos para aperfeiçoar os sistemas de prevenção de inundações e resposta a essas emergências^1,3^.

Ademais, a magnitude e extensão da emergência em saúde pública indica uma sobrecarga significativa para os serviços de saúde e assistência social locais. Diversas unidades, incluindo hospitais, Unidades de Pronto Atendimento (UPA) e Unidades Básicas de Saúde (UBS) tiveram seus atendimentos parcial ou totalmente interrompidos, não apenas por danos físicos às instalações, mas também pela ausência de profissionais de saúde diretamente afetados pelas enchentes^16,17^. A desarticulação dos serviços essenciais compromete a resposta local e regional e reforça a necessidade de incorporar estratégias de resiliência nas políticas de saúde pública. O fortalecimento da capacidade adaptativa do SUS, sobretudo em territórios mais suscetíveis, é fundamental para mitigar os danos e preservar a integralidade do cuidado em contextos de crise.

A continuidade geográfica entre os municípios mais afetados (especialmente nas regiões R1, R2 e R8) sugere que os impactos não tenham ocorrido de forma isolada, mas afetado regiões inteiras de forma interdependente, exigindo respostas articuladas entre municípios vizinhos. A elaboração e implementação de Planos de Adaptação às Mudanças Climáticas e a construção de Planos de Contingência são estratégias essenciais para a redução de riscos e de impactos associados a eventos climáticos extremos e às mudanças climáticas, bem como de desastres ocasionado por elas^5,18,19^.

Esses planos podem fortalecer a capacidade adaptativa das cidades, contribuindo para a prevenção de óbitos, a mitigação de danos à infraestrutura e a redução de perdas materiais. Ao promover ações integradas de preparação e resposta, tais instrumentos são fundamentais para proteger a saúde da população e aumentar a resiliência dos territórios perante os desastres.

Os efeitos de eventos de inundação são, em grande parte, objeto de aproximações e agregações que têm muita imprecisão. Embora a disponibilidade e a qualidade dos dados tenham aumentado e melhorado ao longo do tempo, não há relatos de experiências de um sistema integrado de informações sobre desastres, aumentando o uso de múltiplas fontes de dados em relatórios. Nesse sentido, em muitos eventos a frequência de feridos e afetados é desconhecida, não registradas ou de difícil localização. Para outros resultados, como as mortes, os relatórios também carecem de informação, o que provavelmente contribui para uma subestimação substancial dos impactos de eventos de inundação nas populações humanas^3^.

O uso de indicadores como óbitos, feridos, enfermos, desabrigados e desalojados por habitante permite qualificar o impacto dos desastres e orientar de forma precisa as ações de mitigação, preparação e resposta, além de embasar o planejamento regional de saúde. Entretanto, conhecer as características sociodemográficas dessa população pode contribuir para a organização e o planejamento de ações de recuperação que promovam a equidade.

Um estudo recente avaliou se as ações de resposta durante uma emergência em saúde pública utilizam em suas decisões informações sobre determinantes sociais visando à redução de desigualdades^20^. Os resultados encontrados identificaram três lacunas no processo de decisão e orientação durante a resposta: falta de dados para uma tomada de decisão equitativa, manutenção das desigualdades em saúde existentes e ausência de ferramentas que apoiem a tomada de decisão orientada para a redução das desigualdades^20^.

Dessa maneira, as lacunas identificadas quanto à incorporação de determinantes sociais nas ações de resposta às emergências em saúde pública apresentam estreita relação com os resultados observados no contexto do RS. A elevada proporção de municípios em situação de emergência ou calamidade, somada aos indicadores de mortalidade, adoecimento e deslocamento populacional, reflete desigualdades entre as regiões funcionais, que não podem ser plenamente compreendidas apenas por dados quantitativos de impacto. A insuficiência de informações sociais integradas aos sistemas de monitoramento e tomada de decisão limita a formulação de respostas equitativas e sustentáveis, favorecendo a manutenção das desigualdades já existentes. Assim, a incorporação sistemática dos determinantes sociais da saúde ao planejamento e à gestão das emergências mostra-se essencial para que políticas públicas territorializadas consigam não apenas reduzir os efeitos imediatos dos desastres, mas também enfrentar, de forma estruturada, as disparidades que amplificam seus impactos.

Estudos recentes têm demonstrado que as emergências em saúde pública por inundações provavelmente aumentarão nos próximos anos^3,7^, sendo, portanto, necessário maior foco nas estratégias de registro de informações desses eventos. Isso visa não apenas aprimorar futuras respostas às emergências com base nas lições aprendidas, mas também gerenciar de forma mais rápida e eficaz as informações durante a ocorrência do evento, com foco na equidade. A fragmentação das informações nos sistemas ou a falta de capacidade para captar dados estratégicos em desastres destacam a necessidade de enfrentar esse desafio para os próximos anos no Brasil.

O fato de países com maiores recursos serem capazes de prever e responder melhor a eventos de inundação iminentes^2^ evidencia que o desenvolvimento de sistemas de informação e capacidade de detecção e resposta oportunas a esses desastres em países menos desenvolvidos deve se tornar uma prioridade. As mudanças nas características socioeconômicas, demográficas, geológicas e climatológicas atestam não apenas que as inundações se tornarão mais frequentes, bem como terão impactos mais significativos nas populações humanas nas próximas décadas^1,3,7^.

Por fim, as diferenças entre as regiões funcionais com relação à magnitude dos impactos reforçam a necessidade de políticas públicas territorializadas, com ênfase na prevenção, resposta rápida e fortalecimento da infraestrutura de saúde e proteção social nas áreas mais vulneráveis. Isso exige, de forma urgente, uma governança mais forte no que tange aos riscos de desastres, com estratégias mais eficazes de mitigação de inundações, sistemas de emergência aprimorados e apoio internacional, especialmente para a reconstrução das áreas afetadas após a ocorrência de desastres. Além disso, faz-se necessária a inclusão de estratégias integradas que considerem os efeitos das mudanças climáticas e da degradação ambiental sobre a saúde coletiva, reforçando a importância de uma abordagem proativa e sustentável na gestão de riscos de desastres.

Diante dos impactos observados, torna-se evidente que as emergências em saúde pública provocadas por inundações exigem uma mudança de paradigma na gestão do risco e na formulação de políticas públicas no Brasil. Nesse contexto, os eventos analisados devem ser situados na crise ecológica global, que intensifica a frequência e a gravidade de desastres naturais, impondo desafios crescentes à saúde coletiva e à resiliência do SUS. Mais do que fortalecer estruturas existentes, é necessário avançar na construção de uma cultura de prevenção, com foco na preparação, na alocação adequada de recursos e na atuação coordenada entre os diferentes níveis de governo. Ademais, a integração da vigilância em desastres à agenda de saúde pública, considerando-se dimensões de justiça ambiental e equidade, pode ampliar a efetividade das ações de prevenção e resposta e reduzir desigualdades territoriais no acesso à proteção e ao cuidado.

Cabe ressaltar que a abordagem ecológica adotada neste estudo, embora permita identificar padrões espaciais relevantes e subsidiar o planejamento de ações em saúde coletiva, apresenta limitações, especialmente no que se refere à avaliação de determinantes individuais da vulnerabilidade e às desigualdades socioeconômicas dentro das regiões analisadas. A resposta eficaz a desastres deve estar ancorada em sistemas de informação qualificados, capazes de orientar decisões em tempo real e reduzir desigualdades no acesso à proteção e ao cuidado. A vulnerabilidade exposta por esta emergência reforça que a saúde pública deve assumir papel central na agenda climática, articulando ações de curto, médio e longo prazo para garantir a continuidade dos serviços de saúde e promover justiça social em contextos de crise.
